# Heart Rate Variability and Pain: A Systematic Review

**DOI:** 10.3390/brainsci12020153

**Published:** 2022-01-24

**Authors:** Giuseppe Forte, Giovanna Troisi, Mariella Pazzaglia, Vilfredo De Pascalis, Maria Casagrande

**Affiliations:** 1 Department of Psychology, “Sapienza” University of Rome, 00185 Rome, Italy; mariella.pazzaglia@uniroma1.it (M.P.); vilfredo.depascalis@uniroma1.it (V.D.P.); 2 Body and Action Lab, IRCCS Fondazione Santa Lucia, Via Ardeatina 306, 00179 Rome, Italy; 3 Department of Clinical and Dynamic Psychology and Health Studies, “Sapienza” University of Rome, 00185 Rome, Italy; troisi.1862006@studenti.uniroma1.it

**Keywords:** pain, heart rate variability, autonomic response

## Abstract

Background and Objective: Heart rate variability (HRV) as an index of the autonomic nervous system appears to be related to reactivity to experimental pain stimuli. HRV could better explain the contributions of sympathetic and parasympathetic activity response to nociceptive stimulation. The aim of this study was to systematically review and synthesize the current evidence on HRV in relation to the experience of pain in experimental tasks. Databases and Data Treatment: Studies indexed in the PubMed, PsycINFO, MEDLINE, WebOfScience, and Scopus databases were reviewed for eligibility. Studies on the autonomic response (i.e., HRV) to experimentally induced pain in healthy adults were included. Different methods of pain induction were considered (e.g., thermal, pressure, and electrical). Data were synthesized considering the association between HRV and both pain induction and subjective measures of pain. Results: Seventy-one studies were included. The results underline significant change in both the sympathetic and parasympathetic autonomic nervous systems during the painful stimulation independent of the pain induction method. The autonomic reaction to pain could be affected by several factors, such as sex, age, body mass index, breathing patterns, the intensity of the stimulation, and the affective state. Moreover, an association between the autonomic nervous system and the subjective experience of pain was found. Higher parasympathetic activity was associated with better self-regulation capacities and, accordingly, a higher pain inhibition capacity. Conclusions: HRV appears to be a helpful marker to evaluate nociceptive response in experimentally induced pain. Future studies are also needed in clinical samples to understand better the interindividual changes of autonomic response due to pain stimuli.

## 1. Introduction

Pain is defined as an aversive sensory and emotional experience typically caused by (or resembling that caused by) actual or potential tissue injury [1,2]. Accordingly, it is highlighted that (1) pain is always a subjective experience influenced by biological, psychological, and social factors (differently from nociception) and should be accepted and respected as such, (2) individuals learn the concept of pain through their life experiences, (3) there are several behaviors to communicate it aside from verbal description, and (4) it has an adaptive role, but it can have adverse effects on the individual’s well-being [1].

Depending on the duration, pain can be acute or chronic [3]. While acute pain is considered an adaptive signal that prevents danger and guarantees survival [4], chronic (or persistent) pain is defined as having persisted for at least 3 months [4], and it usually matches chronic diseases and non-treated medical pathologies, affecting the individual’s quality of life [5].

Furthermore, pain can be defined as “somatic” when it involves the skin, subcutaneous tissues, bones, muscles, blood vessels, or connective tissues or “visceral” when it affects the internal organs or the linings of the body cavities [5]. The former can arise from thermal, mechanical, and chemical stimuli, while the latter results from distension or prolonged contraction of the smooth muscle wall of the structure [6]. Both types can be elicited in experimental settings (e.g., [7,8]). Currently, self-reporting measures are the most employed method for assessing experimentally induced pain [9]. For example, pain intensity can be measured by adopting visual analogue scales (VASs), numerical rating scales (NRSs) [10], and verbal rating scales (VRSs) [11].

In acute pain, somatic and visceral noxious stimuli excite the nociceptors [12] and are converted into nerve impulses in order to allow the brain to read them and produce the conscious pain sensation [5]. However, nociception is only a component of the pain experience. In fact, pain sensitivity can be affected by many factors, such as mood, affective regulation strategies, and mental disorders [13]. Pain is a stressor, and it has been considered a specific emotion that reflects homeostatic behavioral drive [13,14]. The affective-motivational and the cognitive-evaluative components enhance the individual’s organization of emotional and behavioral responses [5].

A comprehensive framework to investigate how organisms respond and adapt themselves to diverse types of stressors, including pain, is the Neurovisceral Integration Model (NVIM) [15,16,17]. The authors of the NVIM proposed a core set of neural structures, referred to as the Central Autonomic Network (CAN), which provides the ability to continuously assess and prepare the organism for an appropriate response [18]. In the NVIM, heart rate variability (HRV) has been proposed as an index of flexible and adaptive regulation of the nervous system to organize a homeostatic response to environmental requests and which is related to cognitive functions [18,19,20,21,22]. HRV represents the change in the time interval between successive heartbeats. It is considered a sensitive, non-invasive measurement of autonomic input to the heart [23]. It might provide measures of autonomic nervous system activity in both sympathetic (SNS) and parasympathetic (PNS) branches [23]. Since systems involved in autonomic control (such as periaqueductal gray, the insular and anterior cingulate cortices, amygdala, prefrontal cortex, and nucleus of the solitary tract) [24] are strictly connected with those involved in pain perception, HRV can be considered a reliable index of ANS reactivity to nociceptive stimulation [24,25,26].

Furthermore, HRV appears to be an index of baroreflex activity [15,16,17], one of the body’s homeostatic mechanisms that maintains the blood pressure at a constant level. Patients with chronic pain show a reduction in HRV and baroreflex sensibility due to changes in efferent sympathetic and parasympathetic cardiac activity, which shift the balance to a sympathetic tone prevalence related to catecholamine release [27,28].

HRV is also related to endogenous pain modulation (EPM), a relevant factor in chronic pain development and maintenance [29]. In fact, EPM depends on the excitation–inhibition balance, and HRV can be used as an index of the inhibitory processes involved in these neurovisceral networks. Furthermore, EPM and HRV are connected in both the presence and absence of chronic pain. Accordingly, HRV has several advantages in studies investigating the physiological response to nociceptive stimulation [15,16,17].

Several studies focused on pain sensitivity adopted HRV as a measure of autonomic responses. A systematic review on this topic was conducted by Koenig et al. in 2014 [14]. The authors identified 20 studies showing an increase in sympathetic baroreflex activity and a decrease in vagal parasympathetic activity, as reflected by changes in the frequency domain measures of HRV. However, there has been an increasing number of studies about this relationship in recent years. Therefore, analyzing the new studies can be relevant. This paper aims to systematically summarize the achieved results on the relationship between pain and HRV.

## 2. Method

The review process was conducted according to the PRISMA Statement [30,31] to systematically analyze studies on the relationships between HRV and pain within healthy adult samples. The protocol has not been registered.

Research Strategies

A systematic review was conducted by searching articles published in peer-reviewed journals using the PubMed, PsycINFO, MEDLINE, WebOfScience, and Scopus databases. The last research was conducted on 5 January 2021.

The search was restricted to publications published since 1996 (i.e., years of publication of the first guidelines on the standards of measurements, physiological interpretation, and clinical use of HRV (Task Force of the European Society of Cardiology and the North American Society of Pacing and Electrophysiology, hereafter referred to as Task Force, 1996). Articles focused on analyzing the association between pain and HRV were considered for inclusion. The search strategy used the following keywords: “pain”; “pain sensitivity”; “Heart Rate Variability”; “HRV”; and “IBI”. The reference list of all included studies was screened for additional study citations.

Eligibility Criteria

The list of potential articles produced by systematic research was screened for eligibility. Studies that included one or more methods of experimental induction of pain and the measurement of HRV were selected. Studies that adopted at least one measure of subjective pain perception (e.g., pain thresholds) were judged as eligible. Studies that included participants with medical conditions which could potentially influence this relationship were excluded (e.g., chronic pain disorder, hypertension, and cancer survivors).

Study Selection

**Figure 1:**
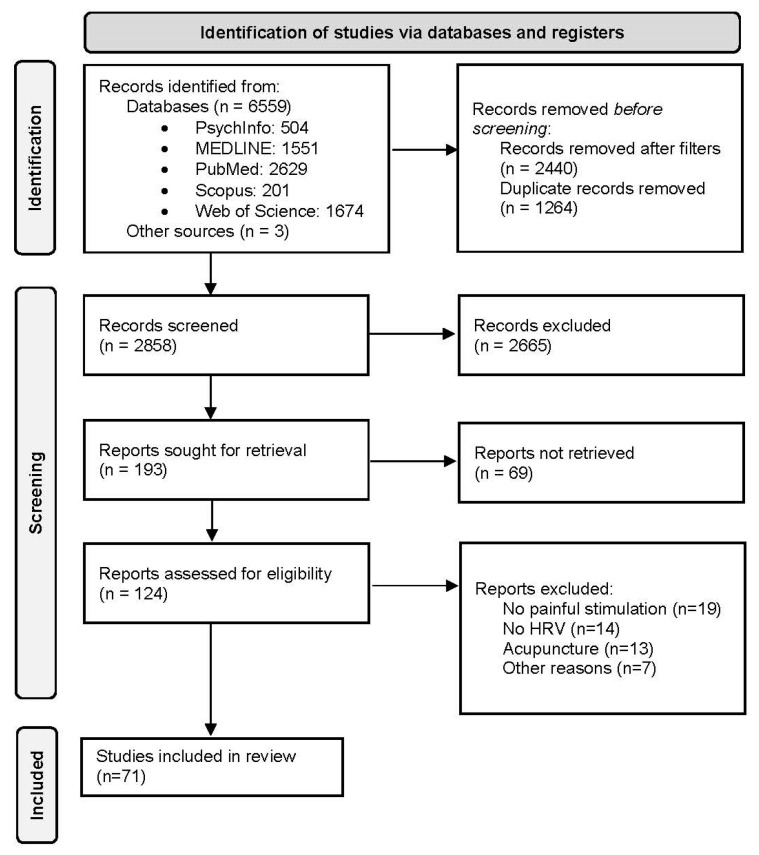
PRISMA flow diagram.

The initial search identified 6559 results imported to the Mendeley database. The screening was performed in two phases. After removing duplicates, the initial eligibility assessment was based on titles and abstracts. Two authors (G.T. and G.F.) independently examined the full texts to confirm the suitability of the studies for the following qualitative synthesis. Then, the full texts that fit the inclusion criteria were screened for the eligibility criteria. Finally, 71 studies were included in the review. During the whole process, disagreements were resolved by consulting a supervisor (M.C.). The selection processes are reported in Figure 1.

Data Collection and Quality Assessment

According to the PICOS approach [30], the following information was extracted from each selected study: (1) author(s) and year of publication, (2) country, (3) sample size and female and male distribution, (4) age of participants, (5) method of pain induction, (6) pain assessment, (7) the main focus of the study, (8) derived HRV measures, and (9) findings. The data are reported in Table 1. 

## 3. Results

Demographic Characteristics of the Sample

The selected studies were conducted from 2004 [59] to 2020 [32], including a total sample of 6364 participants with percentages of 55.7% females and 44.3% males. In three studies, the percentage or the number of women and men was not reported [38,51,61]. Some studies adopted a sample that included exclusively female [39,40,49,50,74,82,84,93] or male participants [57,58,59,62,64,77,78,89,90]. The average age of the participants ranged from 18.98 [52] to 73.44 years [41,42]. The largest range was 18–84 [41].

The selected studies were conducted in Europe (N = 38), the United States (N = 12), Israel (N = 5), Canada (N = 5), Australia (N = 4), India (N=1), Taiwan (N = 1), Brazil (N = 1), China (N = 2), Japan (N = 1), and Korea (N = 1). Except for a study conducted by Kim et al. in 2019 [65], all studies were conducted with homogeneous samples for ethnicity and nationality. All studies adopted a cross-sectional design.

HRV Measurement

In all studies, HRV measurement was conducted by a continuous ECG recording, which lasted at least 5 min as recommended by the guidelines of the European Society of Cardiology and the North American society [99]. Heart rate variability was evaluated considering time domain analyses, frequency domain analyses, or both (see Table 1).

Effects of Different Methods of Pain Induction on HRV

Thermal Stimuli (N = 51)

Thermal stimuli (i.e., cold and heat) were the most adopted method of pain induction (e.g., [49]).

Cold Pain

Cold pain was elicited using mostly the cold pressor task [32,39,43,51,52,56,61,67,78,93], but it was also induced using a cold plate [13,88], a thermal aluminum cylinder device [83], the immersion of a hand [41,57,87,94] or a foot [72,73] in cold water, facial cooling [62], thermal stimulation devices [60,72,91,98], the cold cup test [50], and holding a plastic bottle with iced water [68,92].

Cold pain stimulation elicited an increase in the parasympathetic components of HRV, such as RMSSD [32,67,78,83,87], SDNN [67,83], CVI [51,87], HF [83], mean RR [83], and IBI [62]. Nevertheless, an increase in the sympathetic activity was registered by the reduction in HRV [41], mean IBI [78], and mean RR [61] and the increase in the log LF [60,83,87] and LF/HF ratio [87,88]. A study by De Pascalis and Scacchia [50] found a negative correlation between pain and the time domain but not between pain and the frequency domain. Bendixen et al. [39] found only a reduction in vagal measures (RMSSD, HF power; CCV-HF). However, a relationship between cold pain and the HRV parameters was not found in two studies [56,93].

Heat Pain

Thermal stimulation devices [24,29,33,36,37,45,48,54,57,58,63,65,70,71,72,73,74,77,80,83,91,95,96,98], a laser [47], an IC thermostat [97], and the immersion of a hand in hot water [88] were adopted to induce heat stimulation.

Although no correlations between pain and HRV were reported [53,65,80], other authors underlined an association [70,96]. Both sympathetic and parasympathetic activity changes due to the heat stimuli were reported. On the one hand, an increase in sympathetic activity [48] expressed by the LF/HF ratio [36,47,54] and LF [47] and a decrease in RR [47], lnSDNN [71], and HF [95] was evidenced. On the other hand, a parasympathetic increase was found [74], indexed by the increase in HF [33] and RMSSD [85] and the decrease in lnLF [71]. Aslaksen and Flaten [37] showed that placebo administration before painful exposure reduced the LF/HF ratio after the painful heat stimulation, suggesting that placebo administration can affect the pain experience, reducing physiological stress [37].

Mechanical Stimuli (N = 18)

Pressure pain was elicited by digital pressure algometers [33,38,42,49,57,65,78,79,81,82,84,91], pressing the nail bed with a spring-loaded device [76], and inflating a pressure cuff on the lower leg [86]. Some studies [81,84,86] found higher sympathetic activity during pain, indexed by the decrease in SDNN [84] and HF and the increase in LF and the LF/HF ratio. On the contrary, other authors showed predominant parasympathetic activity in responses to a painful pressure stimulus [38,49,73]. Finally, two studies reported no relationship between pressure pain and the HRV parameters [42,65].

Four studies adopted pinprick stimuli in order to induce experimental mechanical pain, using a set of probes [65] or a von Frey filament [72,73,74,83]. A relationship between parasympathetic activity and pain perception was evidenced.

Electrical Stimuli (N = 9)

Electrical stimulation was induced via electrical stimulators [35,46,49,56,64,80]. In two studies [89,90], sural nerve stimulation was delivered via solid gel surface electrodes. Courtois et al. [49] found an increase in RMSSD during pain while the participants practiced slow deep breathing. Ghione et al. [59] induced pain by exposing participants to an electromagnetic field. The LF component increased during both sham and magnetic exposure, while the HF component remained constant during real exposure but increased during the sham condition. A reduction in HRV was found in two studies [46,64]. Similarly, sympathetic activation increased when the pain was elicited both during mental arithmetic stress and at rest [89,90]. Piovesan et al. [80] registered an increase in the HF components. Finally, the authors of [56] found no relationship between pain and HRV.

Injection of Hypertonic Saline Solutions (N = 5)

A sterile hypertonic saline solution (5%) was injected to induce experimental masseter muscle pain [39,40] and deep and superficial pain [44], and a hypertonic saline solution (7%) [55,66] was injected to induce experimental muscle pain. Two studies found an increase in sympathetic activity during the infusion [55] in both deep and superficial pain [44]. However, parasympathetic parameters such as RMSSD and HF were higher when the only pain stimulation was the solution injection, rather than a condition in which muscle pain was associated with a CPT [39] or with a PASAT [40]. Only one study found no correlation between pain and HRV [66].

Visceral Pain: Esophageal Balloon Distension (N = 2)

Two studies focused on visceral pain [8,76] while adopting esophageal painful balloon distension. Interestingly, one study [8] found that the participants that were classified as “neurotic-introvert” had an increased parasympathetic activity expressed by CVCna in response to pain, while participants classified as “extrovert-emotionally stable” had a high resting CVCna and withdrawal from it during pain stimulation. Others [76] found that both the sympathetic and parasympathetic branches were activated by visceral and somatic pain.

Bed of Nails (N = 1)

One study elicited a painful sensation by letting participants lie on a bed of nails. Stimulation consisted of a soft cotton fabric case filled with a foam rubber rectangle [75]. The authors found an increase in parasympathetic activity expressed by HF when participants were lying on the bed of nails.

Relationship between Subjective Pain Measures and HRV

Subjective Pain Measurement

Subjective measures of pain perception, such as pain intensity, pain unpleasantness, pain thresholds, and pain tolerance, were assessed (see Table 1).

Pain thresholds were assessed by adopting different methods. Regarding heat stimuli, pain thresholds were assessed by increasing the temperature of the device until the subject perceived the stimulus as painful [24,29,45,58,63,65,72,73,74,85,88,91,94,95,96,98] or considered the temperature estimated as painful at a specific point on a VAS. Then, the results were averaged for the number of trials [13,47,53,77] with the calculated thresholds being the average temperature (in °C) at which each participant indicated experiencing noticeable pain and moderate pain during each of the three exposures. Cold pain thresholds were identified as the cold temperature at which the subject reported the stimulus as painful [87,88,91,98] or the total time from immersion until a participant verbally reported pain [67]. Pressure pain thresholds were reached when the participant’s sensation changed from pressure (evoked via a pressure algometer) to pain, which was averaged for a specific number of trials [34,42,65,76,78,79,81,82,91]. The pain thresholds for the electrical stimulation were obtained when the subject reported a pain sensation evoked by the current [35,46,89,90]. The first pain sensation evoked by the balloon distension was defined as the visceral pain threshold [8,76].

Pain tolerance was assessed by considering when the level of heat [45,58,85,96], cold [87,94], pressure [76,78,81,82], or visceral pain [8,76] became unbearable for the participant. Another method to assess cold pain tolerance was calculating the total time in seconds that the participant’s hand was completely submerged in the water [52,67].

Relationship with HRV

The relationship between the subjective pain measures and HRV was reported [24,52,81]. Two studies highlighted a positive relationship between heat pain thresholds and resting LF [13,24]. A higher LF-HRV was positively correlated to a higher temperature at which the subject started to perceive the heat stimulus as painful. Conversely, pain unpleasantness was lower in higher resting LF-HRV [13]. Pain tolerance was associated with higher parasympathetic activity expressed by the HRV (i.e., increase in HF-HRV and decrease in the LF/HF HRV ratio) [52,82].

Recent studies found a negative correlation between parasympathetic activity and the pain intensity ratings [57,68,92]. Accordingly, slow deep breathing and HR biofeedback [45] could reduce both the pain intensity and sympathetic activity expressed by a higher SDNN [45]. Furthermore, placebo analgesia produced an increase in the time domains of HRV and greater pain relief [50]. Pain intensity was also negatively correlated with HRV when the participants had greater parasympathetic activity during the recovery phase [51]. Parasympathetic activity correlated with a more efficient pain modulation capacity [72,73,74].

No correlation between subjective pain measures and HRV was reported or found by many studies (see Table 1).

## 4. Discussion

The main aim of this study was to investigate the relationship between pain and heart rate variability, summarizing the results of experimental studies that induced pain in healthy adult samples. In recent years, an increasing number of studies have adopted HRV as a physiological index of the organism’s ability to provide a flexible response to stress, such as pain. The Vagal Tank Theory [100], relying on the Neurovisceral Integration Model [17], highlights the role of the vagus nerve in the control of cardiac activity and in goal-directed behavior, as well as in the individual self-regulation ability [100]. The Neurovisceral Integration Model [17,19] assumes that the goal-directed behavior and the self-regulation ability of an organism are structurally and functionally supported by the Central Autonomic Network [18], a complex network of brain structures whose primary output is the cardiac vagal control expressed by HRV. Accordingly, our study confirms a relationship between the autonomic nervous system indexed by HRV and the pain response to nociceptive stimulation. Our findings can be reported in two themes: (1) how the autonomic nervous system reacts to pain and (2) how the autonomic nervous system is associated with subjective pain perception.

For the first issue, generally, the studies included in the systematic review reported a significant change in HRV during pain induction. The main finding about the autonomic response to pain is an increase in sympathetic activity (e.g., [36,54]), according to the previous review conducted by Koenig et al. [14]. Evidence suggests that this response is independent of the adopted method of pain induction. Burton et al. [44] found that both deep and cutaneous pain elicit an increase in the LF/HF ratio, in addition to Chouchou et al. [47] underlining a sympathetic activation to heat pain during sleep. The same sympathetic increase was found in response to cold pain, (e.g., [39,60]).

Nevertheless, different circumstances can increase the vagal activity expressed by the parasympathetic components of HRV [45]. For example, different techniques of breathing, such as slow deep breathing [49] or meditation, could reduce vagal withdrawal during pain. Adler-Neal et al. [33] focused on the autonomic responses to pain while mindfulness meditation or sham meditation were practiced. They found a parasympathetic increase during both meditation techniques, possibly due to the slow breathing. Similar results were obtained by Chalaye et al. [45], where slow deep breathing patterns increased vagal activity compared with normal breathing. Cotton et al. [48] found that yoga practitioners for at least 6 years had the same parasympathetic activity during a warm stimulation and a painful one compared with the control group, which reported a withdrawal of vagal activity during the painful stimulation, despite similar pain ratings.

Another aspect that could influence an autonomic response to pain is a drug or medication assumption. Some studies reported that the administration of substances with analgesic effects, such as propranolol [78] or clonidine [73], can increase parasympathetic activity during pain. However, a placebo [50] also generates a similar response. The findings suggest that placebo analgesia, induced administering a placebo, can increase HRV and induce pain relief [37,50], consistent with the Neurovisceral Integration Model [19,25]. Furthermore, autonomic responses to the same painful stimulation can differ depending on the affective states [32] or the empathetic or unempathetic context [53,54].

Another result evidenced by the review is a parasympathetic activation during painful stimulations. Activation of both the sympathetic and parasympathetic branches of the autonomic nervous system appears to be counterintuitive, but the Vagal Tank Theory [100] can explain it. The autonomic nervous system’s reaction to painful stimulation is complex. A large or small vagal withdrawal depends on the level of activity required by the situation and how much top-down executive processing is needed to face the situation [100]. Moreover, the autonomic reaction to pain could be affected by several factors, such as ethnicity, sex, age, body mass index, breathing patterns, the intensity of the stimulation, or the affective state that could influence results [24].

Regarding the second issue, an association between the autonomic nervous system and the subjective experience of pain was found [68]. Higher parasympathetic activity is associated with better self-regulation capacities and, accordingly, a higher pain inhibition capacity [50,52,68]. In this field, an interesting result was related to self-compassion abilities [68,92] that appear to be associated with high HF and lower pain ratings. Tian et al. [92] explained that self-compassion means to treat oneself with kindness and acceptance, and it seems that this ability enhances a better bodily control over pain-related arousal and a better subjective pain experience [92].

Most studies have underlined that when the parasympathetic component of HRV is high, pain relief or a better management of painful situations (e.g., pain tolerance) occur. The inhibitory vagal effect on pain could be responsible for these results. This hypothesis is in line with the Neurovisceral Integration Model, and it is supported by the strong positive relationship between vagal activation and the prefrontal cortex, as highlighted by Perlaki et al. [77]. Finally, Appelhans and Luecken [13] found a negative association between the sympathetic activity expressed by the LF components and pain sensitivity but not between HF and pain. According to the authors, this finding is explicable because LF has a complex association with the arterial baroreflex, a homeostatic mechanism mediated by the autonomic nervous system, whose components also mediate an endogenous pain inhibitory pathway [13]. These results are consistent with a recent study [60] that identified a three-way relationship between HRV, cortical regions underpinning pain processing, and subjective pain experience. In particular, the connectivity between the periaqueductal gray (PAG) [18,25] and the ventromedial prefrontal cortex (vmPFC) at rest was associated with high LF during painful stimulation and lower pain ratings. The PAG receives nociceptive inputs and is involved in descending nociceptive modulation [18,60].

Despite the important update to the study of Koenig et al. [14] and the new evidence reported, this review has some limitations. The strict selection criteria excluded works that may contain relevant information about the activity of ANS in response to pain. Many of the studies included were conducted by the same group of authors, and this could have introduced biases influenced by the theoretical background of those who led the study. However, the selected studies may well represent the state of the knowledge. Moreover, the lack of quantitative analysis (i.e., meta-analysis) lowers the strength of our inferences by not furnishing an effect size for the studies. Another limitation could be indirectly linked to the publication bias. The choice to include only academic articles published in peer-reviewed journals may have limited the selection to only those studies that have obtained results in line with the literature. As a consequence, the results may overestimate this relationship. Moreover, the choice to select only studies published in English could have led to the elimination of studies conducted on other populations and written in different languages, further limiting the generalizability of the results. Finally, the lack of an unambiguous subjective measure of pain sensitivity makes the results heterogeneous. Further studies could develop an instrument to measure pain sensitivity and better define constructs related to pain perception. For example, it could be better to assess pain intensity separately from pain unpleasantness in order to assess the sensory and emotional components more precisely and relate how each component interacts with autonomic activity. Moreover, the role of cognitive functions such as inhibition could be evaluated, considering its association with both HRV and pain.

## 5. Conclusions

According to our results, we can conclude that HRV is a good measure of autonomic reactivity to nociceptive stimulation. Studies that have investigated changes in HRV in response to pain reported changes in autonomic response, both in sympathetic and parasympathetic branches. Our summarized evidence may be helpful for further research and have important clinical implications. Since HRV appears to be impaired in several chronic pain conditions that can worsen the quality of life, future researchers can take advantage of the use of HRV. According to our results, many practices (e.g., yoga and mindfulness) or drugs can increase vagal activity. For this reason, HRV can be a reliable index to assess the efficacy of treatments on pain management in clinical populations. Moreover, it could be tested if techniques of control over HRV such as HRV biofeedback, which were demonstrated to be effective in improving cognitive functions and stress management [101], can increase pain relief or pain management in clinical samples. Further studies are needed to overcome the limitations and also better understand this relationship in the large variety of health conditions associated both with ANS changes and pain (i.e., chronic pain).

## Figures and Tables

**Table 1:** Characteristics of selected studies.

Author (Year)	Country	N (F/M)	Age Mean (SD), Range	Method of Pain Induction	Pain Assessment	Main Study Focus	Derived HRV Measures	Hrv and Pain-Related Findings
Acevedo et al., (2020) [32]	United States	195 (138/57)	20.3 (2.5)	Thermal pain: cold pressor task	PI on a VRS (0–100)	The attenuating role of positive affect on physiological responses to acute pain.	RMSSD	All conditions had a significant increase in RMSSD in response to the CPT. Participants in the low arousal calm and high arousal excited conditions had a significant greater PNS activation during reactivity.
Adler-Neal et al., (2020) [33]	United States	62 (31/31)	30.53 (1.32)	Thermal pain: heat (thermal stimulator)	PI and PU on a VAS (0–10)	Relationship between the PNS and mindfulness-based pain attenuation	HF	Mindfulness-induced PU reductions were associated with higher HF compared with sham-mindfulness meditation. HF significantly increased during pain stimulation.
Appelhans and Luecken (2008) [13]	United States	59 (37/22)	19.74 (1.83)	Thermal pain: cold plate	PI and PU on a NRS (0–100). PTh	Between-person variability in pain sensitivity	LF HF	PI not predicted by LF or HF. High LF predicted lower PU scores and greater of PTh (notable and moderate). No association between HF and pain measures.
de Araujo et al., (2018) [34]	Brazil	57 (39/18)	22.66 (3.9)	Pressure pain (pressure algometer)	PPTh (palmar digital agometer)	Comparing the effects of two mobilization techniques and a placebo intervention, applied on the thoracic vertebral column on HRV and on PPT in asymptomatic subjects	RR STD HR SDNN RMSSD RR trindex HF nu LF nu LH/HF ratio	No difference between groups in HRV, no difference between groups in PPT, except for a reference point (mobilization SLUMP increases PPT of ipsilateral tibialis compared to mobilization PA).
Arsenault et al., (2013) [35]	Canada	20 (9/11)	26.9 (6.1), 21–42	Transcutaneous electrical stimulation	PI and PTh on a NRS (0–100). Pain Catastrophizing Scale.	The effects of respiration on pain modulation	LF HF	LF power higher during the two slow breathing conditions.
Aslaksen et al., (2007) [36]	Norway	64 (32/32)	23.45 (3.24), 19–40	Thermal pain: heat (thermal stimulator)	PI and PU on a VAS (0–100)	The modulating role of experimenter gender on autonomic pain responses	LF/HF ratio	PI ratings lower for male subject × female experimenter. PU higher in women compared with men. LF/HF ratio increased during pain compared with interstimulus intervals.
Aslaksen and Flaten (2008) [37]	Norway	63 (32/31)	24.25 (5.05), 18–40	Thermal pain: heat (thermal stimulator)	PI and PU on VAS (1–100)	The effects of placebo administration on negative emotions and pain ratings	LF/HF ratio	Lower LF/HF ratio and PI during placebo condition.
Balocchi et al., (2005) [38]	Italy	21	22 (1.3)	Pressure pain (pressure algometer)	PI on a scale (1–10)	The effect of hypnotic susceptibility on heart rate variability, in subjects receiving nociceptive stimulation and suggestion of analgesia	LF HF	In Highs, PI different between PAIN and AN. In Lows, HF significantly increased, and LF decreased during PAIN compared with B1.
Bendixen et al., (2012) [39]	Denmark	16 (16/0)	22.9 (2.4)	Muscle pain: injection of hypertonic saline solution; thermal pain: cold pressor test	PI and PU on a NRS (0–10), pain on palpation (POP) on a NRS (0–100)	The modulating role of CPT and PASAT on muscle pain and autonomic function	Mean RR RMSSD SDNN LF HF	Decreased RMSSD, HF, and CCV-HF during CPT. PI and PU higher in HS1 than HS2 during CPT and PASAT conditions.
Bendixen et al., (2013) [40]	Denmark	16(16/0)	23.6, 20–29	Muscle pain: injection of hypertonic saline solution	PI and PU on a NRS (0–10). Pain on palpation (POP) on a NRS (0–100)	The effect of propranolol on hypertonic saline-evoked pain and autonomic activity during rest and during PASAT	Mean RR SDNN RMSSD LF HF LF/HF ratio	Parasympathetic parameters were increased in propranolol group compared with control group.
Boggero and Segerstrom (2019a) [41]	United States	100 (62/38)	Younger adults: 19.06 (1.81), 18–28; older adults: 73.44 (4.73), 65–84	Thermal pain: hand immersion in cold water	PI on a VRS (0–10)	Strategies employed by younger and older adults in order to maintain the affective well-being after an acute pain	Log HRV	Older adults demonstrated significantly lower HRV than younger adults. No correlations between pain and HRV were reported.
Boggero and Segerstrom (2019b) [42]	United States	240 (122/118)	19.38 (2.39), 18–39	Pressure pain (pressure algometer); thermal: immersion of the non-dominant foot in cold water	PPTh	The relationship between self-regulatory ability and the experience of pain	Log HF	No relationship between pain and HRV was found.
Bourassa et al., (2019) [43]	United States	102 (77/25)	19.1 (1.75)	Thermal pain: cold pressor task	PI on NRS (0–10)	The mediating role of a romantic partner in cardiovascular responses during the cold pressor task	RSA	PI significantly lower in the partner present condition compared with control and mental activation conditions. No significant differences in HRV between conditions.
Burton et al., (2009) [44]	Australia	26 (13/13)	28	Muscle and subdermal pain: injection of sterile hypertonic saline solution	PI on a VAS (0–10)	The effects of deep and superficial pain on muscle sympathetic nerve activity	LF HF LF/HF ratio	Significant increase in LF/HF ratio during both muscle and superficial pain.
Chalaye et al., (2009) [45]	Canada	20 (9/11)	25.1 (5.6)	Thermal pain: heat (thermal stimulator)	PTh; PTo.	The effects of breathing on heat pain and autonomic cardiac activity	SDNN LF power HF power	SDNN and LF power significantly increased during pain in deep breathing and HR Biofeedback conditions. No significant differences in HF power. PTh significantly higher during slow deep breathing, HR Biofeedback and distraction conditions; PTo higher in slow deep breathing and HR Biofeedback conditions.
Cho (2019) [46]	Korea	45 (21/24)	22.4 (1.49)	Electrical stimuli	PTh	The effects of electrical stimulation on the autonomic nervous system	HRV	HRV significantly different between the HF-Li and LF-Hi groups immediately after stimulation and between the HF-LI and LF-Hi groups 30 min after stimulation.
Chouchou et al., (2011) [47]	France	14 (4/10)	32.8 (7.3)	Thermal pain: heat (laser)	PTh on a Likert-type scale (0–10)	The assessment of autonomic responses to pain during sleep	Mean RR Wavelet power coefficient of LF, HF, and LF/HF ratio	RR significantly decreased after the stimuli. LF and LF/HF ratio significantly increased after the stimuli. No significant differences in HF.
Cotton et al., (2018) [48]	United States	34 (26/8)	43.18 (11.68)	Thermal pain: heat (thermal stimulator)	PI on a VAS (0–200)PU on a VAS (−100–100)	Autonomic responses to pain in yoga practitioners compared to a control group	RSA SDRR RMSSD pNN50	Yogis had significantly slower RSA during baseline compared with controls. Controls had lower RSA during pain than during warm trials. Yogis had the same lever of RSA during both pain and warm trials.
Courtois et al., (2020) [49]	Belgium	Ex 1: 31(31/0); Ex 2: 28 (28/0); Ex 3: 24 (24/0)	Ex 1: 22.45 (3.10); Ex 2: 20.25 (2.50); Ex 3: 22.55 (3.16)	Ex 1: electrical pain; Ex 2: Thermal (thermal stmulator); Ex 3: Mechanical pain (pressure algometer)	PTh. PI on a NRS (0–10)	The effect of slow deep breathing (SDB) on pain sensitivity, HRV, and baroreflex sensitivity	RMSSD	RMSSD increased during SDB in all experimental conditions. No differences in pain ratings were found, nor in relationships between subjective pain and HRV.
De Pascalis and Scacchia (2019) [50]	Italy	65 (65/0)	24.5 (2.5), 18–36	Thermal pain: cold cup test	Pain expectation and PI on a NRS (0–100)	The influence of personality traits on placebo analgesia	RR SDNN LF power HF power LF/HF ratio	Negative correlation was found between pain and time domain but not between pain and frequency domain.
Dodo and Hashimoto (2017) [51]	Japan	74	21.14 (2.93)	Thermal pain: cold pressor test	Pain perception on the Wong–Baker Faces Pain Rating Scale, PI on a scale (0–5)	The relationship between anxiety sensitivity and autonomic responses during pain	CVI CSI	CVI: significantly higher during CPT in both the low-AS and the high-AS group; low group also higher in recovery compared with rest; during recovery, significantly higher in low-As group than in the high-As group. Subjective pain higher in high-As group than low-As group post-CPT.
Evans et al., (2014) [52]	United States	63 (29/34)	18.98 (1.62)	Thermal pain: cold pressor task	PTo (total time in sec)	The effects of brief mindfulness instructions on pain tolerance and HRV	Log HF power	Higher HRV at baseline positively correlated with greater PTo in the control group.
Fauchon et al., (2017) [53]	France	40 (20/20)	23.2 (8.2)	Thermal pain: heat (thermal stimulator)	PI on a VAS (0–100); PTh	The effect of perceived support on pain modulation and associated vegetative reactions	IBI	No correlation between IBI and pain.
Fauchon et al., (2018) [54]	France	76 (17/59)	27.8 (6.3)	Thermal pain: heat (thermal stimulator)	n.r.	The role of context in the autonomic responses to acute pain	LF HF LF/HF ratio	LF/HF ratio significantly increased in response to pain only during unempathetic condition. Higher LF during unempathetic condition than in neutral condition.
Fazalbhoy et al., (2012) [55]	Australia	12 (1/11)	18–48	Muscle pain: injection of a hypertonic solution	PI on a VAS (0–10); McGill Pain Questionnaire	The cardiovascular responses to tonic pain	LF HF LF/HF ratio	Increasing MSNA group: significantly higher LF power and LF/HF ratio, lower HF power and RMSSD.
Fidanza et al., (2017) [56]	Italy	51 (28/23)	20–27	Electrical stimulation; thermal pain: cold pressor test	PI (0–10)	The relationship between pain modulation (suggestion of analgesia VS Diffuse Noxious Inhibitory Control) and hypnotizability	LF/HF ratio	HRV was not modulated by pain experience.
Geisler et al., (2020) [57]	Germany	33 (0/33)	27.4 (5.65)	Thermal pain: heat (thermal stimulator); pressure pain (pressure algometer); thermal pain: hand immersion in cold water	PI of all stimuli on a VAS (0–100)	Differences in endogenous pain modulation in a sample of athletes and nonathletes	RMSSD SDNN	Athletes had higher RMSSD at rest compared with nonathletes. Negative association between HRV and placebo.
Geva et al., (2017) [58]	Israel	25 (0/25)	35.9 (10)	Thermal pain: heat (thermal stimulator)	PTh and Pto with the thermal stimulator; PI on a VAS (0–10)	Loss of pain modulation under acute psychosocial stress in triathletes	HRV	HRV correlated negatively with the reduction in CPM due to stress.
Ghione et al., (2004) [59]	Italy	10 (0/10)	41 (7)	Electromagnetic field exposure	PTh; PTo	The effects of an electromagnetic field on pain perception and on cardiovascular parameters	LF HF	HF progressively increased during sham exposure and remained constant during magnetic exposure. LF increased during both types of exposure.
Hohenschurz-Schmidt et al., (2020) [60]	United Kingdom	21 (8/13)	26.1 (5.2)	Thermal pain: cold (thermal stimulator)	PI and PU on a VAS (0–100)	Exploration of the neural regions underpinning the relationship between ANS and pain	Log LF Log HF LF/HF ratio	Log LF increased from baseline to cold pain. No associations between HRV and PI. During pain, a positive association was found between log LF and the functional connectivity between dACC and vmPFC. Stronger baseline PAG-vmPFC connectivity had a positive correlation with log LF and a negative correlation with PI.
Huggins and Rakobowchuk (2019) [61]	Canada	16	18–35	Thermal pain: cold pressor test	n.r.	The utility of lacrimal car uncle infrared thermography as a method to monitor alteration in autonomic activity	SDNN RMSSD Mean Rri	Mean RRi decreased with both CPT and MCR.
Iorfino et al., (2016) [62]	Canada	25 (0/25)	23.96 (2.19), 20–30	Thermal pain: facial cooling	PI on a VAS	The role of the vagus in social cognition	HRV	HRV was significantly higher during FC than during NFC; HRV significantly greater during baseline than during RMET.
Jafari et al., (2020) [63]	Belgium	48 (35/13)	22.5 (3)	Thermal pain: heat (thermal stimulator)	PI on a computerized NRS (0–100); PTh	The effects of instructed breathing patterns on experimental pain	Mean IBI RMSSD	Mean IBI lower in SB, SDB-H, and SDB-L conditions compared with UB condition. RMSSD higher in both SDB conditions compared with UB and SB conditions.
Jess et al., (2016) [64]	Germany	20 (0/20)	24.2 (1.9)	Electrical pain	PI on a NRS (0–10)	The evaluation of pain using the Analgesia Nociception Index (ANI) as a measure of HRV	HRV (ANI)	HRV (ANI) scores lower after each stimulus, with a significant drop within the first 2 min after each stimulus.
Kim et al., (2019) [65]	United States	3159 (1810/1349)	26.07 (6.51)	Pressure pain (pressure algometer); mechanical cutaneous pain; thermal pain: heat (thermal stimulator)	Pressure: PTh; mechanical: PTh and PI; heat: PTh, PTo, and PI	The effects of psychological status and cardiovascular responsiveness to racial and ethnic differences in pain sensitivity	SDNN RMSSD VLF LF HF	No correlations between HRV and pain have been reported.
Kobuch et al., (2015) [66]	Australia	50 (25/25)	22.3 (1.15), 18–39	Muscle pain: injection of hypertonic saline solution	PI on a linear potentiometer calibrated to the NRS (0–10); McGill Pain Questionnaire	The relationship between baseline physiological parameters and MSNA responses to tonic muscle pain	LF HF LF/HF ratio RMSSD	No correlations between pain and HRV have been found.
Kostantinou et al., (2020) [67]	Cyprus	43 (37/6)	21.37 (3.72)	Thermal pain: cold pressor task	PI on a VAS (0–10); PTo and PTh	Comparing psychophysiological data captured by wearable and stationary devices during experimentally induced pain	RMSSD pNN50 mean RR SDNN	Both devices registered an increase in SDNN and RMSSD and a decrease in mean RR during experimental phases. Only the wearable devices registered increased pNN50 during experimental phases.
Luo et al., (2020) [68]	China	29 (14/15)	19.93 (1.6), 19–27	Thermal pain: cold (holding a bottle with iced water)	PI on a scale (0–10)	The role and mechanisms of self-compassion in pain perception	HF	Increased HFs were associated with lower PI in the self-compassion compared with control condition. HF higher in self-compassion compared with control.
Martin et al., (2012) [69]	United States	30 (20/10)	21 (5.5)	Electric stimulation	PI on a NRS (0–100); PTh	The influence of experimentally manipulated breathing on pain	Mean RR RMSSD	HRV changed during breathing manipulation, but it was not correlated with pain outcomes.
Matthewson et al., (2019) [70]	United States	84 (42/42)	27.9 (6.29)	Thermal stimuli: heat (thermal stimulator)	PI on a NRS (0–100)	The role of cognitive self-regulation in pain experience and its effects on autonomic responses	IBI	Association was found between IBI and pain.
Meeuse et al., (2013) [71]	Netherlands	73 (44/29)	30 (11)	Thermal pain: heat (thermal stimulator)	PI on a VAS (0–100)	The usefulness of HRV in quantifying pain intensity	IBI lnSDNN lnLF lnHF LF/HF ratio	lnSDNN and lnLF significantly decreased during pain compared with baseline. No significant correlation between PI and HRV parameter was found.
Nahman-Averbuch et al., (2016a) [72]	Israel	40 (20/20)	26.45 (3.85)	Thermal pain: heat (thermal stimulator) and cold (immersion of a foot in cold water); mechanical pain	PTh; PI on a NPS (0–100)	Sex differences in the relationship between pain perception and HRV	RMSSD LF HF LF/HF ratio	Women: LFnu significantly lower and Hfnu significantly higher. Men: higher RMSSD significantly negatively correlated with higher pain adaptation and with more efficient CPM response.
Nahman-Averbuch et al., (2016b) [73]	Israel	40 (20/20)	26.45 (3.85)	Thermal pain: heat (thermal stimulator) and cold (immersion of a foot in cold water); mechanical pain	M Pain: PI on a NPS (0–100); PTh CPM: PI of the TS on a COVAS; CS on an NPS	The effects of oral clonidine on pain perception	RMSSD LF nu HF nu LF/HF ratio	Higher RMSSD in clonidine group. No differences found in the other HRV parameters.
Nahman-Averbuch et al., (2016c) [74]	Israel	30 (30/0)	25.3 (4.1)	Thermal pain: heat (thermal stimulator and the immersion of a hand in hot water); mechanical pain	Thermal: PTh; PI on a NPS (0–100); mechanincal: PI on a NPS (0–100)	Effect of anxiety level on parasympathetic function and pain perception	RMSSD LF nu HF nu LF/HF ratio	Increased parasympathetic activity during recovery in both groups. In the high-anxiety group, higher RMSSD during baseline correlated with higher pain ratings during tonic pain stimulus.
Olsson and von Schéele (2011) [75]	Sweden	32 (20/12)	39.7 (8.6)	Bed of nails	PI on a NRS (0–10)	Subjective physiologic responses of lying on a bed of nails (BN)	SDNN log LF log HF log	HF higher on the BN. Higher SDNN and LF during relaxing instruction on CD while lying on the BN.
Paine et al., (2009a) [8]	United Kingdom	19 (11/8)	22–54	Visceral pain: esophageal balloon distension	PTo; PTh	The relationship between personality and autonomic responses to visceral pain	CVC CSI	CSI increased during pain; no changes in CVC during pain.
Paine et al., (2009b) [76]	United Kingdom	18 (16/2)	35.4 (2.7)	Visceral pain: proximal and distal balloon distension; somatic pain: nail-bed stimulation	PI and PU on a VRS (0–10); PTo and PTh	The relationship between autonomic control and personality in response to visceral and somatic pain	CVT CSI	Increased CVT in the 90 s post-stimulus compared with pre-stimulus CVT. Significantly greater increase in CVT for distal balloon than for nail bed.
Perlaki et al., (2015) [77]	Hungary	18 (0/18)	22.89 (1.96)	Thermal pain: heat (thermal stimulator)	PI on a VAS (0–10); PTh	Investigating the brain structures responsible for pain-related autonomic changes	LF HF LF/HF ratio	The median COPE of left MPFC showed negative correlations with LF/HF ratio and a positive correlation with HFnu. The median COPE of right MPFC showed significant negative correlations with SDNN.
Petersen et al., (2018) [78]	Denmark	25 (0/25)	25.6, 20–37	Pressure pain (pressure algometer); thermal pain: heat (thermal stimulator) and cold (cold pressor test)	PI on a VAS (0–10); PTh; PTo	The effect of propranolol on HRV and pain perception	Mean IBI RMSSD pNN50	Mean IBI significantly lower and RMSSD significantly higher during CPT compared with baseline.
Picchiottino et al., (2020) [79]	France	41 (22/19)	19.9 (3.5)	Pressure pain (pressure algometer)	PTh	The effect of spinal manipulation on cardiovascular autonomic activity and the relationship to pressure pain threshold	LF HF LF/HF ratio RMSSD SDNN	Weak and moderate positive association between changes in PTh and changes in log LF.
Piovesan et al., (2019) [80]	United Kingdom	40 (30/10)	26.2 (3.91)	Electrical pain; thermal pain: heat (thermal stimulator)	PI on a NRS (0–10)	The relationship between autonomic nervous system and perceived duration of pain experience	HF nu	Only high-intensity stimuli were associated with changes in HRV. No relationship between heat pain and HRV was found.
Pollatos et al., (2012a) [81]	Germany (?)	60 (30/30)	24.4 (3.2)	Pressure pain (pressure algometer)	PTh; PTo; PI and PU on a VAS (1–9)	The role of interception sensitivity on cutaneous pain perception	LF HF LF/HF ratio	HFnu significantly decreased while LFnu and LF/HF ratio significantly increased during pain.
Pollatos et al., (2012b) [82]	Germany	22 (22/0)	24.4 (2.8), 21–31	Pressure pain (pressure algometer)	PTh, PTo; PI and PU on a scale (1–9)	The effects of food deprivation on pain perception	HF nu LF/HF ratio	Day 1: PTo positively correlated with HF nu and inversely correlated with LF/HF ratio. Experimental group: after 24 h of food deprivation, significant positive correlation between differences in HF and PTh (hungry minus breakfast).
Poulsen et al., (2019) [83]	Denmark	20 (10/10)	25.0 (4.0)	Capsaicin application; somatosensory functions; thermal pain: heat and cold (thermal stimulator); mechanical pain (calibrated von Frey nylon filaments)	PI on a NRS (0–100)	The region-specific effects of painful stimulation	Mean RR SDNN RMSSD LF HF	Higher mean RR, increased RMSSD, SDNN, LF power, HF power, and CCV-HF power during capsaicin stimulation.
Santarcangelo et al., (2008) [84]	Italy	19 (19/0)	21	Pressure pain (pressure algometer)	PI on a scale (0–10)	Differences due to hypnotizability in the pain-related modulation of HRV during suggestion of analgesia	Mean RR HF LF LF/HF SDNN RMSSD CSI	Mean RR shorter during pain and AN than during baseline. SDNN shorter during pain than during baseline.
Schneider (2020) [85]	Germany	40 (20/20)	35.1, 24–55	Thermal pain: heat (hot immersion test)	PTo, PI, and PU on an NRS (0–10)	The effects of essential oil inhaler on pain perception	RMSSD SDNN	RMSSD: significantly higher during pain than during baseline; higher in the verum condition. SDNN: larger in the verum condition than in the placebo condition.
Sclocco et al., (2016) [86]	United States	11 (3/8)	33 (4)	Pressure pain (pressure cuff)	PI on a scale (0–100)	Investigating specific brainstem nuclei involved in autonomic responses to pain	LF HF LF/HF ratio	HF power decreased during pain compared with rest.
Sharma et al., (2017) [87]	India	30 (15/15)	18–25	Cold pain	PTh, PTo	The modulating role of slow deep breathing on pain perception and cardiac autonomic activity	Mean RR SDNN RMSSD pNN50 LF power HF power LF/HF ratio	PTo, SDNN, RMSSD, LF power, and LF/HF ratio significantly higher during SDB condition compared with spontaneous breathing. HF power significantly lower during SDB condition.
Streff et al., (2010) [88]	Luxemburg	35 (18/17)	24, 19–57	Heat pain; cold pressor trial	PTh; PI on a NRS (0–100); PU on a VAS (0–10)	The physiological effects of two different tonic thermal stimuli	LF/HF ratio	LF/HF ratio relative to baseline higher on CPT compared with HIT.
Terkelsen et al., (2004) [89]	Denmark	26 (0/26)	24, 21–31	Electrical stimulation (sural nerve stimulation)	PTh; PI and PU on an NRS (0–10)	The effects of mental stress on pain perception, HRV, and nociceptive withdrawal reflex	Mean RR SDNN LF HF	Pain + PASAT decreased mean RR, SDNN, LF power, CCV-LF, HF power, and CCV-HF compared with pain at baseline. Pain + attention decreased HF power.
Terkelsen et al., (2005) [90]	Denmark	26 (0/26)	24, 21–31	Electrical stimulation (sural nerve stimulation)	PTh; PI on an NRS (0–10)	The effects of stress on the HRV responses to acute pain	Mean RR SDNN LF HF	Pain at rest: mean RR significantly decreased, LF power and CCV-LF increased. Attention to pain: mean RR decreased and CCV-LF increased. Pain + PASAT: mean RR decreased.
Terkelsen et al., (2008) [91]	Denmark	45 (22/23)	23, 18–27	Cold pain; heat pain; pressure pain	PTh	The effects of the forearm immobilization on pain perception	Mean RR SDNN LF HF	PASAT reduced mean RR, SDNN HF power, and LF power.
Tian et al., (2020) [92]	China	57 (30/27)	20.28 (2.38), 19–33	Cold pain	PI on a scale (0–10)	The impact of the heart rate variability on the relationship between self-compassion and pain	HF	Self-compassion was associated with increased pain when HF was lower; self-compassion was associated with lower pain when HF was higher.
Tousignant-Laflamme and Marchand (2009) [93]	Canada	32 (32/0)	34.3 (7.5)	Cold pressor test	PI on a NRS (0–100)	Autonomic reactivity to pain throughout the menstrual cycle	LF HF LF/HF ratio NN50	No significant differences in HRV were found between rest and CPT.
Tracy et al., (2018a) [24]	Australia	51 (26/25)	21.9, 18–36	Heat pain	PTh	Sex differences in the association between resting HRV and pain sensitivity	lnRMSSD lnLF lnHF	Higher resting LF was associated with higher PTh. In men, significant positive relationship between PTh and resting LF and HF found.
Tracy et al., (2018b) [94]	Germany	35 (29/6)	22.80 (2.45)	Cold pain	PTh; PTo; PI on a VAS (0–10)	The association between HRV and pain sensitivity	LF HF	LF and HF predicted PI.
Treister et al., (2012) [95]	Israel	55 (21/34)	25.9 (4.1), 20–37	Heat pain	PTh; PI on an NPS (0–100)	Comparing different intensities of pain employing different autonomic parameters	HF	HF showed a negative peak (a decrease compared with pre-stimulus) followed by a gradual increase.
Van Den Houte et al., (2018) [29]	Belgium	63 (48/15)	21.49 (3.80), 18–41	Heat pain	PTh; PI on an NRS (0–100)	The association of HRV and negative affectivity in the endogenous pain modulation	RMSSD	Baseline RMSSD significantly related to the difference in PI between the constant and offset condition. Higher RMSSD and larger offset analgesia.
Walter et al., (2014) [96]	Germany	90 (45/45)	18–65	Heat pain	PTh; PTo	The quantification of pain experience using autonomic parameters	IBI	An association between pain and IBI was found.
Ye et al., (2017) [97]	Taiwan	40 (19/21)	22.5, 20–27	Heat pain	PI on an NRS	Changes in physiological parameters during the process of pain production and relief	Mean RR LF HF	LF significantly changed between segments D and E.
Zunhammer et al., (2013) [98]	Germany	20 (10/10)	24.4, 20.7–28.6	Heat and cold pain	PTh; PI and PU on a VAS (0–100)	The relationship between breathing and pain perception	SDRR	All breathing exercises with the exception of paced resting frequency significantly increased SDRR compared with baseline.

*Pain assessment*. PI: pain intensity; PU: pain unpleasantness; PTo: pain tolerance; PTh: pain thresholds; PPTh: pressure pain thresholds; POP: pain on palpation; VAS: visual analogue scale; NRS: numeric rating scale; VRS: verbal rating scale; COVAS: computerized visual analogue scale; CPM: conditioned pain modulation; TS: test stimulus; CS: conditioned stimulus; n.r.: not reported. *HRV measures.* LF: low frequencies; HF: high frequencies; HF nu: normalized units of HF; LF nu: normalized units of LF; SDNN: standard deviation of NN intervals; SDRR: standard deviation of RR intervals; NN50: number of pairs of successive NN (R-R) intervals that differ by more than 50 ms; pNN50: proportion of NN50 divided by the total number of NN (R-R) intervals RMSSD: root mean square of successive differences; IBI: interbeat interval; RSA: respiratory sinus arrhythmia; CVI: cardiac vagal index; CSI: cardiac sympathetic index; CVT: cardiac vagal tone; CVC: cardiac vagal control; ANI: analgesia nociception index.
